# Parental stress in families of children with special educational needs: a systematic review

**DOI:** 10.3389/fpsyt.2023.1198302

**Published:** 2023-08-10

**Authors:** Andrew W. Y. Cheng, Cynthia Y. Y. Lai

**Affiliations:** Department of Rehabilitation Sciences, Faculty of Health and Social Sciences, The Hong Kong Polytechnic University, Kowloon, Hong Kong SAR, China

**Keywords:** parental stress, special educational needs, parental stress assessment tool, systematic review, coping

## Abstract

**Background:**

This systematic review aimed to identify the risk factors and protective factors for parental stress in families with children with special educational needs. Studies have indicated that the wellbeing of families is related to the physical, psychological, and social conditions of the children, as well as the family functioning, stress coping strategies, and social and professional support of their parents. The parents of children with special educational needs experience high levels of parental stress. Identifying the associated risk factors and protective factors may shed light on the provision of interventions to promote the mental wellbeing of these parents.

**Methods:**

Boolean operators were used to search multiple online databases, and the Preferred Reporting Items for Systematic Reviews and Meta-Analyses guidelines were applied in this systematic review. Data were extracted into categories of population, age, region, the child's diagnosis, the stress-measuring instrument, and the risk factors and protective factors.

**Results:**

Twenty-six studies, including 5,169 parents and 3,726 children, were reviewed. The following four major risk factors and protective factors were found to be associated with parental stress: the sex of the parents, diagnosis-related coping issues, socioeconomic characteristics, and social isolation of the parents.

**Conclusions:**

This systematic review identified four significant risk factors and protective factors related to social support from couples, family members, and social circles. Various agencies may provide financial and manpower assistance and professional support and services to improve the parents' knowledge and coping skills, as well as affectional support, early screening, and continuous assessment of the parents' progress. Social policies and interventions offering continuous and diagnosis-related support to the parents of children with special educational needs are highly recommended.

## Introduction

Over 291 million children and adolescents younger than 20 years globally were estimated to have developmental disabilities and special educational needs (1). These children may have disabilities, such as intellectual disability, epilepsy, hearing or vision loss, autism spectrum disorder (ASD), or attention deficit hyperactivity disorder (2). Parenting a child with SEN may be a wonderful journey with a sense of accomplishment and excitement, but it may also be challenging or even impose a caregiving burden (3).

Parents of children with SEN may be prone to psychological distress, such as anxiety, sleep disturbances, and frustration, when facing the behavioral problems of their children (4, 5). For example, a study by Caley (6) found that the mother, as the primary caretaker of SEN experienced higher stress levels. Stress may come from managing the challenging behavior and special needs of children. Aif et al. (7) concluded that parents bringing up children with SEN face overall family life changes, and burdens affect many aspects of life. Prolonged stress from handling children's daily-life problems and diverse and challenging needs may affect family functioning and wellbeing (8, 9). Parents may also feel stress and experience additional financial strain when taking care of their children who have SEN in combination with fluctuating health conditions or the requirement for repeated hospitalization (10–12).

The impact of taking care of children with SEN is multifaceted. It is not uncommon for such parents to have marital problems, physical and psychological distress, or mental health issues (13–15). Studies have provided some hints of the possible factors contributing to the mental wellbeing of the parents of children with SEN. For example, families who receive services designed based on person-centered and family-centered approaches have been found to experience less marital stress. These families may develop more appropriate or adaptive techniques to take care of their children and may feel more supported by healthcare service providers (3). Psychoeducation programs designed to address the challenging behavior and health conditions of children with SEN (e.g., ASD) have been shown to effectively reduce the maternal burden (16).

To improve the wellbeing of the parents or informal caregivers of children with SEN, it is essential to identify the risk factors and protective factors for stress in this population. The aim of this study was to identify the risk factors and protective factors affecting the stress level of the parents of children with SEN. A better understanding of these factors may help to provide guidelines for service providers to design effective interventions (17–19).

## Methods

### Operation definition

This is a systematic review of studies on stress experienced by the parents of children (aged 3–17) with SEN. In this paper, SEN include specific learning difficulties, intellectual disability, ASD, attention deficit hyperactivity disorder, physical disability, visual impairment, hearing impairment, and speech and language impairments (20). Parent-related stress represents the level of dysfunction in the parent–child system related to the parent's functioning (15).

### Article selection

The initial search was first applied to the Cochrane Database of Systematic Reviews to determine if any previous reviews covered our topic. After confirming that there was no equivalent review, an extended search was conducted.

This systematic review followed the Preferred Reporting Items for Systematic Reviews and Meta-Analyses protocols (21). Boolean searches were used to interrogate PubMed, Embase, Cochrane Library, PsycINFO (via ProQuest), and Web of Science databases. Studies published from 1960 to 2021 were searched. The following search terms were entered into the databases: “parental stress,” “special educational needs,” “risk factor,” and “stress level measurement.” The detailed search terms included “parental stress AND special educational needs,” “parental stress AND special educational needs and/or disabilities,” “parental stress AND special educational needs AND measuring risk level,” “parental stress AND special educational needs AND level of risk,” “parental stress AND special educational needs AND risk factor,” “parental stress AND special educational needs AND stress level measurement,” “parental stress level measurement AND special educational needs,” “parenting stress level measurement AND special educational needs,” and “parent stress level AND special educational needs.”

Articles were included in the analysis if:

- they reported primary research investigating stress experienced by the mother, father, or both parents of children with SEN, with at least one quantitative measurement or in a qualitative format;
- they were published in a peer-reviewed journal; and
- the full-text publication was available in English.

Articles were excluded if:

- they reported a study unrelated to parental stress or
- they reported secondary research or were a conference presentation or unpublished thesis.

**Figure 1:**
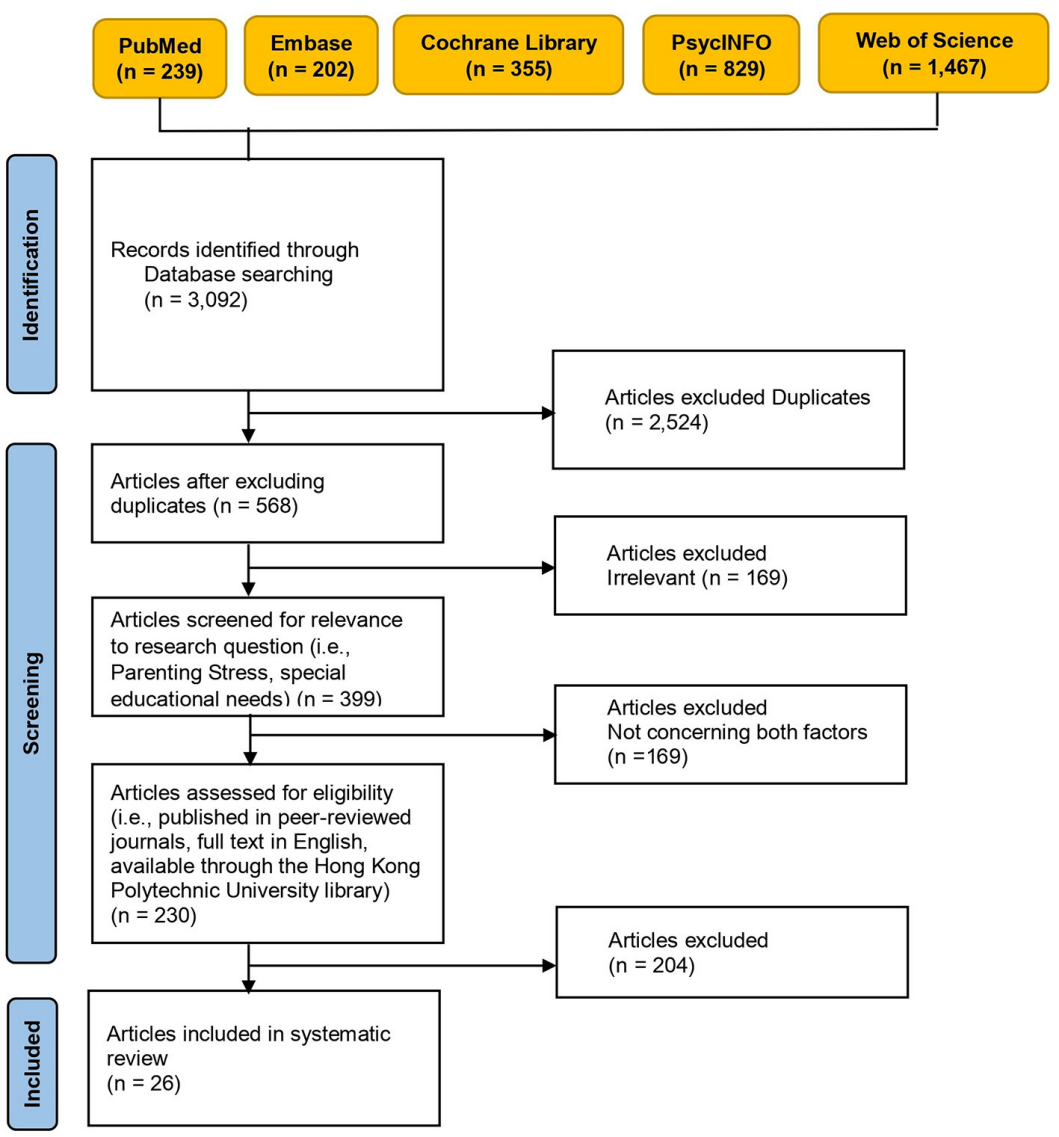
Preferred reporting items for systematic reviews and meta-analyses flow chart of the screening process.

The primary database search identified 3,092 records. Information was imported to EndNote 20 (Clarivate, London, UK) for the deduplication process to eliminate redundant data. After completing this process, 2,524 articles were excluded. In addition, 169 articles were excluded as they were not relevant. Another 169 articles were excluded as they did not evaluate both parental stress and SEN. Another 204 articles were excluded because they reported secondary research, consisted of a manuscript or conference material with an abstract only, or did not report parental stress. No further eligible studies were identified during the manual screening process. Finally, 26 articles were included in this systematic review. The details of the screening process, which followed the Preferred Reporting Items for Systematic Reviews and Meta-Analyses guidelines (22), are described in Figure 1.

## Results

### Description of the articles reviewed

After screening the articles, relevant data were extracted and summarized in tabular format. The characteristics of the included studies are summarized in Table 1.

**Table 1:** Characteristics of the reviewed articles.

**References**	**Population and age**	**Region**	**Diagnosis**	**Stress measurement tools**	**Risk factors/protective factors**
Spratt et al. (23)	- Mother and father total 181 - Children population not mentioned - 4–12 yrs old	USA	- Combined developmental, behavioral, neurological, and emotional problems (DBC) - Intraventricular hemorrhage documented at birth (IVH) - Learning and/or attention problems (LD/ADHD) - Neural tube defects (NTD)	Parenting stress index Short Form (PSI/SF)	Risk factors:- Behavioral problems of child• Protective factors:- Parents such as those with IVH and NTD know from birth that their children might have cognitive difficulties and are able to adapt their expectations and/or coping accordingly
Mu noz-Silva et al. (24)	- Mother 126 - Boy 90, girl 36 - 6–17 yrs old	Spain	ADHD	- Parenting stress index Short Form (PSI/SF) - Stress index for parents of adolescents (SIPA)	Risk factors:- Negative impact on social life - Conduct problems• Protective factors:- Not mentioned
Shaffer et al. (25)	- Mother 11 - Children 15 - Age not mentioned	USA	ADHD/ADD, autism, anxiety, dyspraxia or dysgraphia, dyslexia, speech related disorder, fragile-X syndrome	- Depression Anxiety and Stress Scales (DASS-SS) - The Perceived Stress Scale (PSS) - Structured, open-ended questions to collect qualitative results	Risk factors:- Contrasting perspectives regarding child's needs and proposed solutions (perceive more therapy or medication) - Parents' thoughts and emotions associated with worries about current and future situations - Child's behaviors and special needs - Perceived judgment from others - Educational barriers (limitation for school or educators) - Parent is not engaging in self-care or restorative activities• Protective factors:- Sense of community/Feeling a sense of belonging - Awareness of the need for self-care - Mindfulness-based practices and attitudes (become more aware of your own actions)
Zeng et al. (26)	- Parent 226 - Child 216 - Age not mentioned	China	ASD	Parenting stress index Short Form (PSI/SF)	Risk factors:- Insufficient respite care service - Lack of services to provide emotional health• Protective factors:- Family support on parental stress - Support group for parents to learn from each other
Sarimski et al. (27)	- Mother 115, father 10 - Children population not mentioned - Age not mentioned	Germany	Intellectual disabilities, hearing impairment or visual impairment	Parenting stress index Short Form (PSI/SF)	Risk factors:- Child behavioral problems - Category of disability: more stress in intellectual disability, than visual impairment, than deaf. Determined by a heterogeneous group of variables including child disability itself• Protective factors:- Actively participate in therapeutic activities - Sufficient professional support - Family support
Roccella et al. (28)	- Parent 330 - Boy 173, girl 157 - 5.8–11.6 yrs old	Italy	Primary monosymptomatic nocturnal enuresis (PMNE)	Parenting stress index Short Form (PSI/SF)	Risk factors:- Characteristic of diagnosis: uncontrollable and unpredictable situation - Mother reported more stress than father• Protective factors: - Psychological support on emotional dimensions from professional.
Feizi et al. (29)	- Mother 285 - Children population not mentioned - 6–12 yrs old	Iran	Chronic physical diseases (diabetes, epilepsy, renal problems, and leukemia), difficult sensory- motor and intellectual problems (blindness, deafness, intellectual disability, cerebral palsy), psychological disorder (scolionophobia, autism, ADHD, conduct disorder, oppositional defiant disorder, and learning disability)	- Parenting stress index Short Form (PSI/SF) - Mean Mother = 116, father = 106	Risk factors: - More stress on mother of children with sensory-motor mental and chronic physical problems, than psychological disorders• Protective factors:- Professional support on diagnostic knowledge and professional consultation on stress management
Obeidat (30)	- Mother 53, father 45 - Children population not mentioned - 4–11 yrs old	Jordan	Type 1 diabetes mellitus	Parenting stress index Short Form (PSI/SF)	Risk factors:- Mother reported more stress than fathers - Increasing age of parents - Challenges of caring for lifelong illness• Protective factors:- Professional support through educational program in terms of disease management - Psychological support from nurse
Leyser and Dekel (31)	Family 82, mother 77, father 26	Israel	Learning disabled, intellectual disability, organic disability (impairments to central nervous system), autistic, developmentally delayed, physically impaired	Structured interview	Risk factors:- Financial problems - Support from community - Feelings of stigma - Time available for special child and siblings - Unacceptable behaviors of disabled, need for continued care, lack of time for themselves• Protective factors:- Support from health care professionals (counseling, training on knowledge, practical skills, in-depth family therapy) - Maintain close contacts with extended family members (e.g., own parents)
Park and Yoon (32)	- Mother 5 - Children population not mentioned - Age not mentioned	Korean	Deaf	Qualitative research, one-to-one, in-depth, semi structured interviews with participants in their native Korean language	Risk factors:- Frustration with parenting their child - Struggling between mainstream education and special education - Continuing to be alienated from mainstream education settings - Feeling left out and hurt in family relationships - Making a sacrifice for the child - Change in values of life - Importance of services meeting parents' needs• Protective factors: - Physician had provided support in regard to the psychological and emotional aspects, and detailed information on approaches to handling deafness
Chan et al. (33)	- Mother 560, father 150 - Boy 615, girl 95 - School age	HK	Polymorbidity: language disabilities, ASD, ADHD, specific learning disability, intellectual disability	Parenting stress index Short Form (PSI/SF) Chinese version	Risk factors:- Developmental polymorbidity condition brings higher level of parenting stress, physical and emotional strain, child problem behaviors and emotional symptoms give up valued activities• Protective factors:- Not mentioned
Shin and Nhan (34)	- Mother 225 - Boy 135, girl 65	Vietnam	Cognitive delay	Parenting stress index Short Form (PSI/SF) Likert response categories simplified to 1–3	Risk factors:- Having a child of cognitive delay, mother's education—fewer coping strategies, father's health condition—financial burden, economic status—lack of resources• Protective factors: - Social support
Weitlauf et al. (35)	- Mother 70 - Children population not mentioned - Age not mentioned	USA	ASD	Parenting stress index Short Form (PSI/SF)	Risk factors:- Child challenging behavior - Autism symptoms or cognitive abilities - Less positive marital relationship exacerbate the impact of parenting stress• Protective factors:- Positive martial relationship buffer the impacts of parenting stress
Gerstein et al. (36)	- Family 115 - 3–5 yrs old	USA	Intellectual disabilities (ID)	Parenting daily hassles (PDH)—to measure daily parenting stress	Risk factors:- Mothers experienced higher stress than fathers• Protective factors:- Not mentioned
Chu et al. (37)	- Parent 110 mother 80, father 30 - Children population not mentioned - Age not mentioned	Malaysian	ASD	- Affiliate Stigma Scale - Caregiver Burden Inventory	Risk factors:- Without assistance in caring of child - Time-demanding tasks - Relational problems with the child• Protective factors:- Do not possess negative views about themselves - Malays are found to be the most supportive and less rejecting of individuals with mental disorders - Formal professional support such as therapy, or informal support - Parents adopt positive meanings to their experience in order to repossess a sense of control over their lives as a means to buffer their stress
Chan and Mo (38)	- Mother 18, father 2 - Children 20 - 7–12 yrs old	China	Dyslexia alone, or with ADHD	In-depth interview	Risk factors:- Conditions come with diagnosis, daily responsibilities and maternal role inequalities, social comparisons and promoting a parent-blaming culture, competitive school culture with high expectations on academic result and heavy homework load• Protective factors: - Placing more value on children's “other strength”, cultural and religious beliefs to reframe current situation, active communication with teachers, enhance support from spouses
Arif et al. (7)	- Mother 415, father 32, - School age 150	Pakistan	Mild, moderate, and severe disability from three special schools	Coping Strategies Inventory (CSI)	Risk factors:- Parents who perceive the child's disability as a burden or some sort of tension face a high level of stress and they mostly blame themselves for the child's disability• Protective factors:- Parents who perceive the presence of disability in their family as a challenge experience lower level of stress
Hadadian (39)	- Family 15 - Boy 8, girl 7 - 20–48 months old	USA	Developmental delay, physically or sensory impaired (from early childhood special education programs)	- Parent Stress Index-−101 items - Parental questionnaire	Risk factors:- Children's behavior and temperament - Stress level of mother = father• Protective factors:- Mothers who receive support from their spouses (fathers as a source of support) - Parents' strength and needs were recognized and supported by professional communities
Duvdevany and Abboud (40)	- Mother 100 - Boy 54, girl 45 - Age 4–12 years old	Israel	Intellectual disability (ID)	- Emotional Stress Perception Scale - Arabic version	Risk factors:- Self-disclosure of family information and needs when approaching government service, as a source of shame and conflict with family• Protective factors:- Strong family support by relatives as in traditional Israeli Arab society
Macias et al. (41)	- Boy 99, girl 71 - Age 4–12 years old	USA	Children with Special Health Care Needs (CCSHCN): Neural tube defects (NTD), Developmental-behavioral disabilities (DBD), or history of Perinatal intraventricular hemorrhage (IVH)	- Parenting stress index Short Form (PSI/SF)	Risk factors: - Children have toileting problem• Protective factors:- Clinicians in primary care settings provide assessment and referral - Professional support like counseling or other psychological services - Clinicians provide ongoing stress appraisals to monitor progress
Jones (42)	Parent 13	UK	Recognized physical or intellectual disability	Questionnaire for feedback, non-standardized tool	Risk factors:- Not mentioned• Protective factors:- Adopt stress management skills from profession, in group format in terms of counseling, Rational Emotive Therapy, hypnosis and time management
De Clercq et al. (43)	- Mother 415, father 32 - Children 447 - School age 6–17	Belgium	Autism spectrum disorder, cerebral palsy, Down syndrome	- Parenting Stress Index (PSI 40 items) - Dutch version	Risk factors:- Role restriction, marital stress, attachment stress, competence stress, feelings of social isolation• Protective factors:- Not mentioned
Ren et al. (44)	- Mother 1,049, father 402 - Children 1,451 - Preschool 97 - School age 1,354	China	Autism, intellectual disability, hearing impairment, visual impairment	- State Anxiety Inventory (S-AI) - Parenting Stress Index—Short Form-15 (PSI-SF-15) - NEO Five-Factor Inventory (NEO-FFI) - Multidimensional Scale of Perceived Social Support (MSPSS)	Risk factors: - Family's socioeconomic level declines, not able to go to school normally during epidemic, lack of professional support• Protective factors:- College education or above, monthly family income above 15,000 CNY, higher material wellbeing, job satisfaction, and family satisfaction
Huang et al. (45)	- Mother 80 - Children 80 - Pre-school 65, School-age 15	China	Autism spectrum disorder (ASD), including low and high functioning ASD	Parenting stress index Short Form (PSI/SF)	Risk factors:- Financial issue, such as cost of interventions and treatment for children with ASD is often not covered by insurance• Protective factors:- Social supports: better knowledge of behavior management through both education and receipts of ASD services, multidisciplinary parent education program designed for caregivers
Masulani-Mwale et al. (46)	Parents 170	Malawi, Africa	Intellectual disabilities	Self-Reporting Questionnaire (SRQ)	Risk factors:- Lower socioeconomic status, single motherhood, perceived burden of care, low confidence in managing the disabled child, lack of psychosocial support, knowledge of child's disability and having more than one child with a disability in the family• Protective factors:- Not mentioned
Li et al. (47)	- Mother 20, father 2 - Children 25	USA	Autism spectrum disorder (ASD)	Parenting stress index Short Form (PSI/SF)	Risk factors:- Autism diagnosis, challenging child behaviors, stigma and marginalization, and financial burden• Protective factors:- Behavioral interventions for stress management with particular focus on their parenting role

This review covered 26 studies, which included seven studies from the USA; five from Europe; and 14 from Asia, including five from China and Hong Kong. Of these 26 studies, 11 involved ASD, six involved ADHD, and 11 involved intellectual disability. For the articles involving intellectual disability, different terms, such as cognitive delay (34), intellectual disability (30, 31), and mental disability (42), were applied.

### Risk factors and protective factors

The following four major categories of risk factors and protective factors were identified: the sex of the parents, diagnosis-related coping issues, socioeconomic characteristics, and social isolation of the parents.

### Sex of the parents

Mothers were reported to perceive more parental stress than fathers in four studies, mainly due to their labor workload and their need to handle unpredictable situations (28–30, 36). Only one study reported the same stress level between mothers and fathers in 15 families (39). Parental stress was found to increase with the increasing age of the parents (29).

### Diagnosis-related coping issues

Twelve studies reported that the major risk factors were the challenging behavior or maladaptive behavior of children with SEN. Behavioral problems related to ASD or ADHD or the emotional problems of children with SEN were related to higher levels of parental stress (23–25, 27, 28, 31, 33, 35, 39, 47). Higher levels of emotional problems of SEN children, higher parental stress were recorded. Parents who do not have adequate professional or social support may develop poor parenting practices. Negative coping habits or handling methods may result from the lack of appropriate knowledge and competence to handle stressful situations (24, 31, 32, 43).

Protective factors, such as professional support (23, 25, 27–32, 34, 35, 41, 44), and positive coping skills and techniques, such as stress management strategies, were related to lower levels of parental stress (31, 42, 45, 47). That is, a better understanding of the child's progress and gaining knowledge and skills to manage the behaviors of children with SEN were also protective factors.

Furthermore, a study of parents reported that those who knew the diagnosis of their child since birth were able to adjust their expectations (23). For example, the parents were able to cope better if the cognitive deficits of their children were identified at birth, rather than acquired later.

### Socioeconomic characteristics

Having financial problems (31, 34, 45) and a family's lower socioeconomic level (44, 46) were related to higher levels of parental stress in parents of children with SEN compared with parents of typically developing children.

Five studies (31, 34, 44–46) from countries including Israel, Vietnam, Malawi of Africa, and China reported that financial issues were a risk factor, but parents demonstrated hesitation and resistance to seek financial assistance due to social pressure and associated feelings of shame about disclosing the family's situation. Another two studies found that the self-disclosure of the family's information and needs when approaching government services was a source of shame and conflict with other family members.

### Social isolation of parents

Five studies reported that social isolation and a poor social life were sources of parenting burdens and increased the stress level of parents (24, 31–33, 43). This occurred because the lack time for oneself and insufficient social support may increase perceived levels of stress. Another three studies reported that poor family support induced extra stress during the caregiving journey (37, 38, 43). Four studies indicated that stigma and judgment from others induced stress in the parents of children with SEN (25, 31, 40, 47).

However, it was found that supportive family and marital relationships and sufficient labor help were protective factors. Support from professionals (25, 27–29, 31, 32, 37, 41, 42, 45, 47) and family members (7, 26, 27, 29, 44) were factors contributing to a reduction in the levels of parental stress. Regarding family support, four studies showed that positive marital relationships and support from spouses were essential protective factors (35, 38, 39, 44). Three studies conducted in the Asian region: Jordan, Malaysia, and Israel (30, 37, 40) indicated that strong family support promoted a positive perception of the parental task as a challenge rather than a burden. Sufficient professional services were found to be useful for improving the mindfulness and attitudes of the parents (25) and their knowledge of disease and behavioral management (29, 32, 45, 47). Ongoing assessments of the parenting situation (41) and stress management techniques (30, 42, 47) may also reduce parental stress. Thirteen studies applied the Parenting Stress Index–Short Form to evaluate parental stress.

## Discussion

### Sex of the parents

The results indicated that there were differences in parental stress levels between fathers and mothers, with mothers perceiving higher stress levels than fathers. A higher stress level may be induced by closer interactions with children to handle behavior related to their diagnosis. Role identification within the family may be one of the underlying reasons for this finding; that is, the father may focus on breadwinning rather than caring or daily house activities. A heavy workload for the daily care of children and more frequent interactions with problematic behavior were some of the sources of parental stress. The major caring role was shouldered by the mother, leading to higher stress levels for mothers. Studies (48, 49) have shown that parental stress is induced by heavy physical and psychological burdens related to daily caring and continuous worrying about the child's future. This may have negative effects on the entire family system and directly impact the quality of life of the whole family (50–52). In China, mothers generally take greater responsibility for childcare than fathers (45). Mothers face unique challenges in handling SEN children's emotional and behavioral symptoms. The mothers may perceive being left alone in the caring duties as they play a significant role in raising SEN children. When adequate external support is available, the mothers may gain confidence to adopt a more positive living style. Professional support may include caring techniques, knowledge of SEN, and information on available services. Direct intervention from different professions may reduce parental stress more effectively.

The father was identified as an immeasurable source of support (39), Further studies on how the family role and the father–child relationship affect parental stress are recommended. In the meanwhile, service providers may consider to actively involve father into training as a partner, to fully utilize their strengths to support their spouses. Adequate support to fathers and recognitions afterwards are therefore foundations to development of fathers.

### Diagnosis-related coping issues

For diagnosis-related issues, one of the identified risk factors was handling the challenging behavior of the children. The underlying deficits leading to the challenging behavior of children with SEN are varied. The lack of knowledge and skills in behavioral management of children with SEN may induce negative coping behaviors (e.g., reacting with anger), which cause further anxiety and stress to the parents (6, 53). Therefore, diagnosis-specific psychoeducation programs (e.g., knowledge of the specific conditions of children with SEN and parenting, behavioral management, problem-solving, and stress management skills) would be helpful to reduce the stress levels of the parents (5, 10, 54).

Formal support from professionals and government policy may provide structured and sustainable assistance to parents to improve their knowledge and skills to handle problems or crisis in daily care. The informal support system, on the other hand, including support from family members, relatives and friends, and even neighborhoods, can act as a moderating factor to reduce parental stress and improve the wellbeing of mothers (40). Multiple studies indicated social support is an essential protective factor to reduce perceived parental stress (39, 44, 47). Some parents may consider their social life is being sacrificed. Muñoz-Silva et al. (24) found that the strongest predictor of mothers' stress is the negative impact on their social life, but not the children's emotional problems nor the mothers' perceived social support. Therefore, the parent's social life should be considered when designing an improvement plan for the family.

Because of negative coping habits or handling methods, parents have a feeling of shame when children exhibit uncontrollable challenging behavior in a social environment (40) and they blame themselves (7, 38) or have low confidence in managing their children with SEN (46). Long-term follow-up by a professional is recommended. For example, professionals may provide ongoing monitoring of the parents' stress levels and refer them to suitable professional services as required.

### Socioeconomic characteristics

Financial and human resource constraints may be some of the major risk factors for parental stress, as parents from lower socioeconomic levels need extra support for expenses and manpower to care for their children with SEN (55). For example, the parents of children with SEN may perceive extra physical, time, and financial concerns when bringing their children to receive treatment. To minimize the traveling costs, telehealth or tele-rehabilitation may be alternative options to support the parents. However, further enhancement of telehealth or tele-rehabilitation systems is needed. Government policies on financial support for the parents of children with SEN are also recommended.

### Social isolation of parents

Emotional support was one of the main protective factors identified. Professional staff may provide opportunities for parents to express their concerns, unmet needs, and worries. Social groups were able to provide opportunities for parents to share their unique stories with others with similar experiences. Studies have also suggested the importance of social relationships in reducing parental stress (3, 6).

Chu et al. (37) conducted a study which recruited 110 parents of children with ASD, with 80 mothers and 30 fathers, with ages mainly around 21–50, from an online support group. It was found that although stressful situations and parenting difficulties occurred due to time-demanding tasks required by the children, parents expressed that affiliate stigma does not affect their stress levels and even quality of life. It is worth to note that the participants were active support group members who shared knowledge and offered social support in caring for ASD children. The members of this support group were also exposed to massive amounts of information and experience sharing and also receiving therapy from different professionals. Sharing positive coping strategies can tackle stress associated with feelings of helplessness and isolation (56). This indicates some essential features of peer support group that may help reducing parental stress. Cultural factors should be considered as 93.6% of the participants in that study are Malay. Malay perceives the child as a form of test from God, and parents will be judged in the afterlife (57). This may have an impact on the view of caring duties of caring for their children with SEN.

### Limitations

This systematic review has several limitations. First, most of the research participants were mothers (3,585 mothers and 699 fathers). The participants joined the studies voluntarily, but actively participating attendants may have a more positive attitude, which may have biased the results. Second, not all studies included a control group or applied a randomized control trial in the research design. Third, the screening, selection, and data extraction processes were performed by a single researcher. Therefore, further studies on parental stress experienced by fathers, performed with the involvement of a second researcher are recommended.

## Conclusions

This systematic review synthesized recent findings on the risk factors and protective factors for parental stress in the families of children with SEN. Regarding risk factors, handling the challenging behavior of children is the major component of diagnosis-related coping issues. Financial problems and lower socioeconomic levels restricted the time and resources to care for SEN children. Social isolation of parents is considered as a risk factor caused by poor social life, insufficient family support, and even stigma and judgment from others. On the other hand, protective factors such as professional support can improve positive coping skills and gain a better understanding of a child's progress. Supporting family systems and positive marital relationships can lead to better resilience and coping techniques for parents, leading to better wellbeing.

Healthcare professionals have a unique role in remediating parents' burdens. Parents expected a gracious explanation or time from professionals. Parents treasured being taken by the professionals seriously. Considering the parental stress induced by taking caring of SEN children, healthcare professionals can act as a source of information and direct support as well as assisting parents in developing positive coping techniques. A valid parental stress assessment is essential. It is noticed that the most commonly applied parental measurement tool by healthcare professionals was the Parenting Stress Index-Short Form. Moreover, measurement tool identifying the risk factor and protector factor would be needed to promote the resilience of the parents.

Parenting a child with SEN may be a complicated and demanding task, which may lead to additional financial, physical, psychological, and social burdens. This review identified the possible risk factors and protective factors for parental stress, with the aim of identifying ways to relieve this stress.

The findings from the reviews suggest when identified parents of SEN with high stress, service provider should provide choices of stress-relieving programs, direct assistance in manpower or financial issue, and also provide professional opinion to develop positive handling technique. When existing services cannot fulfill particular needs, referral to health care professionals should be considered, in order to design custom made program. It may guide the practice of service providers. The development of diagnosis-specific parental support programs and future studies on their impact on parents with different family roles are recommended.

## Data availability statement

The original contributions presented in the study are included in the article/supplementary material, further inquiries can be directed to the corresponding author.

## Author contributions

AC and CL designed the review. AC conducted the systematic review and prepared the tables and figures. CL edited the review. All authors contributed to the article and approved the submitted version.
